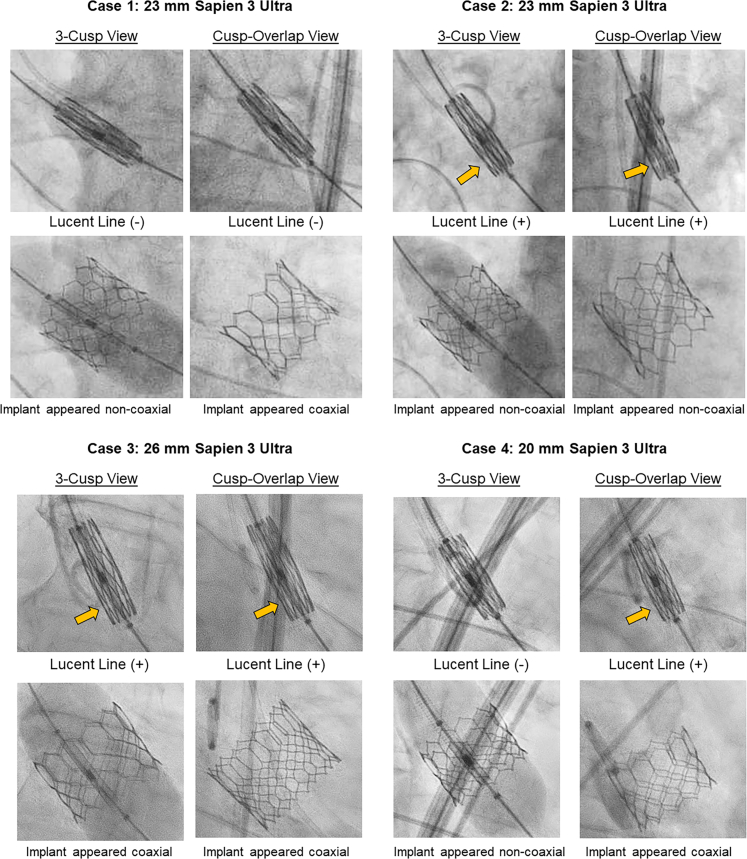# Fluoroscopic “Lucent Line” Visualization in SAPIEN 3 TAVR Deployment: Reproducibility and Impacts on Outcomes

**DOI:** 10.1016/j.jscai.2025.103856

**Published:** 2025-08-19

**Authors:** Parasuram Krishnamoorthy, Manish Vinayak, Negar Salehi, Sahil Khera, Sunny Goel, Amit Hooda, Stamatios Lerakis, Malcolm Anastasius, George D. Dangas, Pedro Moreno, Samin K. Sharma, Annapoorna S. Kini, Gilbert H.L. Tang

**Affiliations:** a Division of Cardiology, Mount Sinai Hospital, New York, New York; b Department of Cardiovascular Surgery, Mount Sinai Hospital, New York, New York

**Keywords:** coaxiality, lucent line, transcatheter aortic valve replacement

## Abstract

**Background:**

Fluoroscopic radiolucent (lucent) line (LL) visualization in SAPIEN 3 (S3) transcatheter aortic valve replacement (TAVR) has been advocated to optimize implant depth, but reproducibility and outcomes remain unknown. Our goal was to determine the incidence of LL seen at conventional deployment view during S3 TAVR and associated outcomes.

**Methods:**

From April 2017 to September 2022, fluoroscopic images of 1130 consecutive transfemoral S3 TAVR were retrospective analyzed. LL visualization at the time of S3 positioning (+ or −) and coaxiality (coaxial [C] or noncoaxial [NC]) of the S3 valve at final implantation in the 3-cusp coplanar view were respectively evaluated. Procedural and in-hospital outcomes per Valve Academic Research Consortium 3 definitions were determined.

**Results:**

LL was present in only 64.8%, and coaxial implant was achieved in only 45.6% of the cases. Three main scenarios were identified: (1) LL(+)/C implant in 44.5%, (2) LL(+)/NC implant in 20.3%, and (3) LL(−)/NC implant in 34.2%. LL deployment resulted in a deeper S3 implantation (LL[+]/C: 19.8% ± 11.0% ventricular vs LL[+]/NC: 18.5% ± 9.7% vs LL[−]/NC: 17.5% ± 12.1%, at noncoronary cusp; *P* = .008; LL[+]/C 16.3 ± 11.5% vs LL[+]/NC: 15.6% ± 11.1% vs LL[−]/NC: 13.9% ± 13.5% at left-coronary cusp; *P* = .02). When comparing the 3 scenarios of S3 deployment, there were no differences in outcomes including paravalvular leak, pacemaker implantation, and hemodynamic performance.

**Conclusions:**

LL visualization in S3 TAVR could not be obtained in a significant portion of cases, did not result in a coaxial valve deployment in a majority of cases, and did not achieve a higher valve implantation. However, no short-term clinical and echocardiographic impact was observed.

## Introduction

With transcatheter aortic valve replacement (TAVR) expanded to lower risk and younger patients, contemporary practice has favored higher valve implantation to avoid new persistent conduction disturbances and pacemaker implantation. TAVR with balloon-expandable valves is performed by positioning and deploying the transcatheter heart valve (THV) at the 3-cusp coplanar view per both conventional practice and manufacturer recommendations. However, to enable higher THV implantation with the latest generation balloon-expandable SAPIEN 3/Ultra (S3) valve (Edwards Lifesciences), the radiolucent (lucent) line (LL) method has recently been proposed to remove the parallax of the S3 valve during positioning to facilitate a more coaxial and higher implant at either the 3-cusp or right anterior oblique (RAO), caudal projection.^1^, ^2^, ^3^, ^4^ However, to visualize the LL, one often needs to change the fluoroscopic C-arm angle away from the conventional coplanar projection, resulting in a valve deployment not coaxial to the annular plane. Although previous studies have shown that the LL deployment of S3 valve resulted in reduced pacemaker implantation, to date, there has been no study that evaluated the impact of LL positioning on the coaxiality of S3 implantation and associated outcomes.^1^, ^2^, ^3^, ^4^ Since the advent of TAVR performed in high-risk and extreme-risk patients under both fluoroscopic and transesophageal echocardiographic guidance, efforts have been made to simplify the procedure to be performed primarily under fluoroscopic guidance, with transthoracic echocardiography (TTE) reserved only to evaluate paravalvular leak (PVL) and procedural complications.^5^ The LL concept was derived to help TAVR implanters identify a consistent fluoroscopic landmark on the S3 valve to achieve a higher implantation in order to reduce pacemaker implantation.^1^, ^2^, ^3^, ^4^ Initially, the concept was presented on social media with S3 implantation at the 3-cusp view. Subsequently, the Cleveland Clinic group used the LL in a RAO, caudal fluoroscopic projection irrespective of the annular plane projection to achieve a higher S3 implantation.^2^ However, this technique is not standard practice among most TAVR centers and is not in accordance to manufacturer recommendations. A recent comparative study between 3-cusp view and cusp-overlap view by the Vancouver group showed a higher S3 implantation achieved with cusp-overlap view.^4^ However, the authors did not comment whether LL was used systematically in cusp-overlap view to aid in higher deployment.

We therefore sought to determine the following retrospectively: (1) the incidence of LL visualized during S3 positioning at the conventional 3-cusp coplanar projection and associated coaxialty of S3 implantation and (2) the impact of coaxiality in S3 implantation on clinical and echocardiographic outcomes.

## Materials and methods

### Patient population

From April 2017 to September 2022, 1130 consecutive patients who underwent S3 TAVR at our institution were retrospectively reviewed. All patients underwent TTE and multidetector computed tomography evaluation to determine candidacy for TAVR and S3 THV sizing.

### Evaluation of LL visualization during S3 positioning and coaxiality after deployment

**Figure 1:**
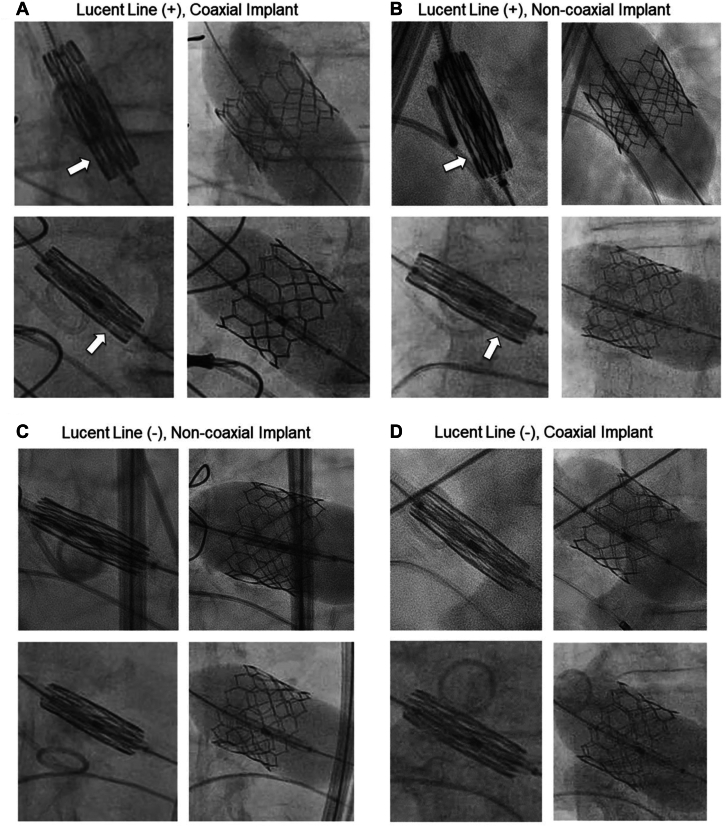
**Definitions of lucent line (LL) and coaxiality during SAPIEN 3/Ultra valve positioning and deployment.** Sample cases on the visualization of LL on the 3-cusp coplanar fluoroscopic view during SAPIEN 3/Ultra valve positioning and subsequent deployment. Four scenarios are possible: (A) LL(+)/coaxial; (B) LL(+)/noncoaxial; (C), LL(−)/noncoaxial; and (D) LL(−)/coaxial (D).

All S3 TAVR procedures were performed per standard practice and manufacturer recommendations (ie, not using LL), under monitored anesthesia care with TTE guidance as default, or general anesthesia with transesophageal echocardiographic guidance when indicated. Standard definition was used to identify all views and conventional deployment technique was used in all cases. In brief, a 3-cusp coplanar fluoroscopic view was determined by placing a pigtail catheter at the base of right coronary cusp.^6^ The S3 THV was positioned across the aortic annulus on the 3-cusp coplanar view, with the bottom of the mid-balloon marker above annular plane depending on the desired target implant depth. During deployment, the pigtail was placed in the noncoronary sinus and withdrawn just before the valve was being deployed. LL was identified as the radiolucent line that is located at the superior aspect of the lowest set of stent struts of the crimped valve. Retrospectively, patients were categorized as LL present (+) or absent (−) depending on LL visualization during THV positioning (Figure 1). The coaxiality of the implanted valve relative to the annular plane was retrospectively determined by visualizing if parallax of the S3 THV frame was present (+) or absent (−) (Figure 1). A coaxial (C) S3 implant was defined as parallax being (−) while a noncoaxial (NC) implant was defined as parallax being (+). Patients were divided into 4 scenarios based on S3 positioning and deployment: (1) LL(+)/C, (2) LL(+)/NC, (3) LL(−)/C; and (4) LL(−)/NC.

Additionally, sensitivity analysis of 50 patients deployed using a dedicated LL view were performed to compare implantation depth and coaxiality with conventional deployment views. A dedicated LL view was typically obtained in RAO caudal projections.

### Procedural characteristics and in-hospital outcomes

Balloon predilation, S3 balloon filling (nominal, overfill, and underfill) for valve deployment, and positioning of the bottom of the mid-balloon marker above the annulus during S3 positioning were performed at the discretion of the procedural heart team, based on anatomic characteristics determined on multidetector computed tomography and visualized on intraprocedural fluoroscopy. When anatomically feasible, a more aortic implant was targeted to avoid new-onset conduction disturbance. Volume of S3 balloon inflation, position of the bottom of the mid-balloon marker during S3 positioning, final S3 THV implant depths at non- and left-coronary cusps, as well as other procedural characteristics, were included in our analysis. In-hospital clinical and echocardiographic outcomes were reported per Valve Academic Research Consortium 3 definitions.^7^ All intraprocedural echocardiographic evaluations were performed by 2 experienced structural heart imagers (S.L., M.A.) involved in all our TAVR procedures. To minimize interobserver variability, the same imager would evaluate both intraprocedural and predischarge TTE on each patient. Details of interventional operators during the study period are included in Supplemental Table S1.

### Statistical analyses

Continuous variables were reported as means with SD, whereas categorical variables are reported as frequencies and proportions. The clinical, anatomic, echocardiographic, and procedural characteristics between the LL groups were compared. Differences between groups were detected using 1-way analysis of variance for continuous variables and χ2 or Fisher exact test for categorical variables. Data were collected by M.V. and N.S., and independent analysis was performed by P.K. and G.H.L.T., and there was no significant observer variability. All statistical tests were 2-tailed with *P* < .05 considered significant. Statistical analyses were performed using SPSS version 24.0 (IBM Corp).

### Ethical statement

Our study was carried out in accordance with the appropriate ethical guidelines. Our study received institutional review board approval and patient consent was waived.

## Results

### Incidence of LL visualization and baseline characteristics

**Figure 2:**
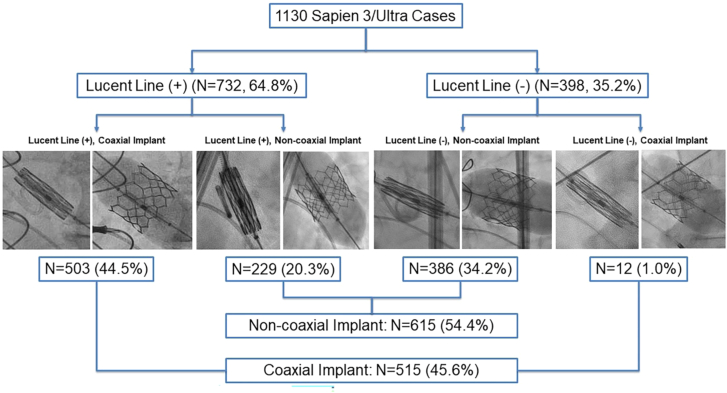
**Incidence of lucent line (LL) visualization and coaxialty of SAPIEN 3/Ultra valve implantation.** Among 1130 SAPIEN 3/Ultra (S3) TAVR cases, the incidence of LL being present and resulting coaxialty of valve implantation was shown. LL was present only in 64.8% of cases, and among them, only 67.8% of cases had coaxial S3 implant.

Among 1130 patients included in our study, the incidence of LL(+) seen during S3 positioning was 64.5% when using the 3-cusp coplanar view, with overall 54.4% of the cases resulting in a noncoaxial S3 implantation (Figure 2). The proportion of the 4 S3 positioning and deployment scenarios were as follows: (1) LL(+)/C implant in 44.5%, (2) LL(+)/NC implant in 20.3%, (3) LL(−)/C in 1.0%, and (4) LL(−)/NC implant in 34.2% (Figure 2). Among the S3 cases where the LL was observed, coaxial implant was achieved in only 68.7%. Because the third scenario LL(−)/C was only 1.0% of our overall cohort, we focused our analyses on the remaining 3 scenarios.

Table 1 lists the baseline patient characteristics. In the LL(−)/NC group, there were more females, patients had higher mean surgical risk scores, more diabetes incidence, and more bicuspid anatomy. Table 2 lists the anatomic characteristics and respective S3 valve size distributions among the 4 groups. There were no differences in the percent oversizing by annular and left ventricular outflow tract (LVOT) areas, annular and LVOT eccentricities, and ≥moderate annular or LVOT calcification. The LL(−)/NC group had more 23 mm S3 but fewer 29 mm S3 implanted.

**Table 1:** Patient characteristics among groups undergoing SAPIEN 3 transcatheter aortic valve replacement.

Characteristic	Total (N = 1130)	LL(+)/C (n = 503)	LL(+)/NC (n = 229)	LL(−)C/NC (n = 386)	*P* (3 groups)^a^	LL(−)/C (n = 12)	*P* (4 groups)^a^
Age, y	79 ± 9	79 ± 8	79 ± 9	80 ± 9	.13	78 ± 9	.20
Female sex	399 (35.3)	159 (31.6)	75 (32.8)	160 (41.5)	.007^a^	5 (41.7)	.017^a^
STS PROM, %	4.4 ± 3.6	4.1 ± 3.2	4.3 ± 3.2	4.9 ± 4.2	.006^a^	4.4 ± 2.2	.017^a^
Heart team risk					.28		.49
Low	173 (15.3)	76 (15.1)	45 (19.7)	51 (13.2)		1 (8.3)	
Intermediate	360 (31.9)	159 (31.6)	71 (31.0)	125 (32.4)		5 (41.7)	
High	356 (31.5)	168 (33.4)	68 (29.7)	116 (30.1)		4 (33.3)	
Extreme	241 (21.3)	100 (19.9)	45 (19.7)	94 (24.4)		2 (16.7)	
Frailty	547 (48.4)	234 (46.5)	107 (46.7)	202 (52.3)	.19	4 (33.3)	.22
CAD	589 (52.1)	262 (52.1)	119 (52.0)	201 (52.1)	>.99	7 (58.3)	.98
Diabetes	371 (32.8)	183 (36.4)	71 (31.0)	111 (28.8)	.047^a^	6 (50.0)	.052
Previous stroke	113 (10.0)	44 (8.7)	28 (12.2)	40 (10.4)	.33	1 (8.3)	.53
CVD	194 (17.2)	88 (17.5)	42 (18.3)	63 (16.3)	.80	1 (8.3)	.77
PVD	202 (17.9)	77 (15.3)	48 (21.0)	76 (19.7)	.10	1 (8.3)	.15
Lung disease	253 (22.4)	109 (21.7)	53 (23.1)	47 (37.3)	.71	3 (25.0)	.92
Atrial fibrillation	307 (27.2)	136 (27.0)	64 (27.9)	107 (27.7)	.96	0 (0.0)	.20
CKD	408 (36.1)	170 (33.8)	86 (37.6)	148 (38.3)	.33	4 (33.3)	.52
Pulmonary hypertension	302 (32.0)	157 (31.2)	73 (31.9)	125 (32.4)	.93	7 (58.3)	.26
Previous pacemaker	111 (9.8)	47 (9.3)	25 (10.9)	37 (9.6)	.79	2 (16.7)	.78
Previous cardiac surgery	168 (14.9)	82 (16.3)	32 (14.0)	50 (13.0)	.36	4 (33.3)	.15
Previous PCI	423 (37.4)	195 (38.8)	89 (38.9)	136 (35.2)	.50	3 (25.0)	.54
Bicuspid anatomy	91 (8.1)	41 (8.2)	10 (4.4)	40 (10.4)	.032^a^	0 (0.0)	.075
NYHA III/IV	1026 (90.8)	462 (91.8)	203 (88.6)	351 (90.9)	.38	10 (83.3)	.43
LVEF ≤ 35%	124 (11.0)	50 (9.9)	23 (10.0)	48 (4.1)	.45	3 (25.0)	.26
Baseline TTE							
Mean gradient, mm Hg	39.9 ± 14.7	38.8 ± 14.2	39.3 ± 12.7	41.6 ± 16.3	.014^a^	40.4 ± 12.4	.035^a^
Peak gradient, mm Hg	65.9 ± 22.0	64.5 ± 21.2	65.2 ± 18.9	68.1 ± 24.6	.047^a^	65.8 ± 17.4	.10
AVA, cm^2^	0.73 ± 0.16	0.74 ± 0.16	0.73 ± 0.16	0.71 ± 0.16	.017^a^	0.64 ± 0.09	.010^a^

Values are expressed as n (%), mean ± SD (range), or median ± IQR.

AVA, aortic valve area; C, coaxial; CAD, coronary artery disease; CKD, chronic kidney disease; COPD, chronic obstructive pulmonary disease; CVD, cerebrovascular disease; LL, lucent line; LVEF, left ventricular ejection fraction; NC, noncoaxial; NYHA, New York Heart Association; PCI, percutaneous coronary intervention; PVD, peripheral vascular disease; STS PROM, Society of Thoracic Surgeons predicted risk of operative mortality; TTE, transthoracic echocardiography; TAVR, transcatheter aortic valve replacement.

a A *P* value of <.05 is significant.

**Table 2:** Anatomic characteristics by computed tomography among LL groups in SAPIEN 3 Ultra transcatheter aortic valve replacement.

Characteristic	Total (N = 1130)	LL(+)/C (n = 503)	LL(+)/NC (n = 229)	LL(−)C/NC (n = 386)	*P* (3 groups)^a^	LL(−)/C (n = 12)	*P* (4 groups)^a^
Annulus							
Maximum diameter, mm	27.7 ± 2.8	27.9 ± 2.8	28.0 ± 3.0	27.3 ± 2.7	.002^a^	26.9 ± 2.4	.005^a^
Minimum diameter, mm	22.6 ± 2.3	22.7 ± 2.2	22.8 ± 2.2	22.3 ± 2.3	.003^a^	23.1 ± 1.9	.008^a^
Average diameter, mm	25.2 ± 2.4	25.3 ± 2.4	25.4 ± 2.4	24.8 ± 2.4	.001^a^	25.0 ± 2.0	.004^a^
Area, mm^2^	496.5 ± 91.9	502.8 ± 92.4	506.1 ± 92.9	482.9 ± 90.0	.001^a^	489.5 ± 75.0	.004^a^
Area-derived diameter, mm	25.0 ± 2.3	25.2 ± 2.3	25.3 ± 2.3	24.7 ± 2.3	.001^a^	24.9 ± 1.9	.003^a^
Perimeter, mm	79.5 ± 7.4	80.0 ± 7.4	80.4 ± 7.3	78.5 ± 7.4	.001^a^	78.7 ± 6.1	.004^a^
Perimeter-derived diameter, mm	25.3 ± 2.4	25.5 ± 2.4	25.6 ± 2.3	25.0 ± 2.3	.001^a^	25.1 ± 2.0	.004^a^
Oversizing by annular area, %	2.2 ± 8.2	2.5 ± 8.3	2.9 ± 8.5	1.5 ± 77	.086	−2.6 ± 6.6	.029^a^
Annular eccentricity, %	18.1 ± 6.2	18.1 ± 5.8	18.1 ± 7.5	18.2 ± 5.6	.97	14.0 ± 5.4	.14
LVOT							
Maximum diameter, mm	28.7 ± 3.3	28.8 ± 3.2	29.1 ± 3.3	28.2 ± 3.4	.001^a^	28.5 ± 2.1	.003^a^
Minimum diameter, mm	21.6 ± 2.9	21.8 ± 2.8	21.8 ± 2.9	21.2 ± 3.0	.007^a^	22.8 ± 2.1	.008^a^
Average diameter, mm	25.1 ± 2.9	25.3 ± 2.8	25.5 ± 2.9	24.7 ± 3.0	.001^a^	25.6 ± 1.9	.003^a^
Area, mm^2^	491.2 ± 116.4	497.9 ± 113.5	502.1 ± 117.3	475.4 ± 119.5	.005^a^	509.8 ± 77.4	.012^a^
Area-derived diameter, mm	24.8 ± 3.0	25.0 ± 3.1	25.1 ± 2.9	24.4 ± 3.0	.006^a^	25.4 ± 2.0	.013^a^
Perimeter, mm	79.8 ± 9.3	80.4 ± 9.0	80.8 ± 9.2	78.5 ± 9.6	.002^a^	81.3 ± 6.5	.005^a^
Perimeter-derived diameter, mm	25.4 ± 3.0	25.5 ± 3.1	25.7 ± 2.9	25.0 ± 3.1	.005^a^	25.9 ± 2.1	.011^a^
Oversizing by LVOT area, %	5.3 ± 15.3	5.9 ± 15.5	5.5 ± 13.7	5.6 ± 16.2	.93	−5.7 ± 12.3	.092
LVOT eccentricity, %	24.4 ± 6.9	24.4 ± 6.8	24.8 ± 7.0	24.6 ± 6.8	.55	2.2 ± 5.9	.12^a^
≥Moderate annular	104 (9.2)	49 (9.7)	16 (7.0)	37 (9.6)	.45	2 (16.7)	.50
LVOT calcification	59 (5.2)	34 (6.8)	8 (3.5)	17 (4.4)	.12	0 (.0)	.22
Valve size, mm					<.001^a^		<.001^a^
20	21 (1.9)	4 (0.8)	2 (0.9)	14 (3.6)		1 (8.3)	
23	350 (31.0)	145 (26.8)	58 (25.3)	144 (37.4)		3 (25.0)	
26	561 (49.6)	252 (50.1)	121 (52.8)	180 (46.6)		8 (66.7)	
29	198 (17.5)	102 (20.3)	48 (21.0)	48 (12.4)		0 (0.0)	

Values are expressed as n (%), mean ± SD (range), or median ± IQR.

C, coaxial; LL, lucent line; LVOT, left ventricular outflow tract; NC, noncoaxial.

a A *P* value of <.05 is significant.

### Procedural characteristics

Table 3 lists the procedural characteristics of the 4 groups. There were overall 2 patients (0.2%) who required placement of a second THV during the procedure, both in the LL(+)/NC group. One was a 23.0-mm S3 THV that was implanted too aortic a position with significant PVL requiring a more ventricular placement of a second 23.0-mm S3. The other was a 26.0-mm S3 Ultra THV implanted in a degenerated 25.0-mm Mosaic surgical aortic valve (Medtronic) but developed significant transvalvular aortic regurgitation after balloon valve fracture with a 26.0-mm true balloon. A 29.0-mm Evolut PRO+ (Medtronic) was implanted inside the 26-mm S3 acutely.

**Table 3:** Procedural characteristics among LL groups in SAPIEN 3 transcatheter aortic valve replacement.

Characteristic	Total (N = 1130)	LL(+)/C (n = 503)	LL(+)/NC (n = 229)	LL(−)C/NC (n = 386)	*P* (3 groups)^a^	LL(−)/C (n = 12)	*P* (4 groups)^a^
Transfemoral	1124 (99.5)	502 (99.8)	224 (97.8)	386 (100.0)	<0.001^a^	12 (100.0)	0.002^a^
Valve-in-valve	29 (2.6)	18 (3.6)	5 (2.2)	5 (1.3)	0.091	1 (8.3)	0.10
Conscious sedation	973 (86.1)	442 (87.9)	200 (87.3)	323 (83.7)	0.17	8 (66.7)	0.051
Predilation	676 (59.8)	296 (58.8)	131 (57.2)	242 (62.7)	0.34	7 (58.3)	0.53
Postdilation	88 (7.8)	39 (7.8)	15 (6.6)	32 (8.3)	0.73	2 (16.7)	0.58
Second valve placed	2 (0.2)	0 (0.0)	2 (0.9)	0 (0.0)	0.020^a^	0 (0.0)	0.048^a^
Coronary obstruction	0 (0.0)	0 (0.0)	0 (0.0)	0 (0.0)	>.99	0 (0.0)	>.99
Annular rupture	5 (0.4)	4 (0.8)	1 (0.4)	0 (0.0)	0.21	0 (0.0)	0.36
Conversion to surgery	2 (0.2)	1 (0.2)	1 (0.4)	0 (0.0)	0.46	0 (0.0)	0.66
Final balloon filling					0.21		0.34
Underfill	156 (13.8)	77 (15.3)	35 (15.3)	43 (11.1)		1 (8.3)	
Nominal	645 (57.1)	293 (58.3)	123 (53.7)	223 (57.8)		6 (50.0)	
Overfill	329 (29.1)	133 (26.4)	71 (31.0)	120 (31.1)		5 (41.7)	
Balloon marker position above annulus, mm	1.5 ± 1.3	1.5 ± 1.4	1.5 ± 1.3	1.5 ± 1.3	0.82	1.4 ± 1.3	0.94
NCC depth, % ventricular	18.8 ± 11.2	19.8 ± 11.0	18.5 ± 9.7	17.5 ± 12.1	0.008^a^	21.7 ± 16.3	0.015^a^
LCC depth, % ventricular	15.3 ± 12.2	16.3 ± 11.5	15.6 ± 11.1	13.9 ± 13.5	0.020^a^	16.7 ± 13.0	0.047^a^
Intraprocedural echocardiogram							
Paravalvular leak, %					0.65		0.51
None/trace	88.5	89.3	90.0	87.3		75.0	
Mild	11.0	9.9	10.0	12.4		25.0	
≥Moderate	0.5	0.8	0.0	0.3		0.0	
Transvalvular AR, %					0.67		0.88
None/trace	99.9	99.8	100.0	100.0		100.0	
Mild	0.1	0.2	0.0	0.0		0.0	
≥Moderate	0.0	0.0	0.0	0.0		0.0	
Mean gradient, mm Hg	4.3 ± 2.3	4.4 ± 2.5	4.2 ± 2.4	4.3 ± 2.0	0.75	6.0 ± 2.7	0.38

Values are expressed as n (%), mean ± SD (range), or median ± IQR.

AR, aortic regurgitation; C, coaxial; LL, lucent line; LCC, left-coronary cusp; NC, noncoaxial; NCC, nonleft coronary cusp.

a A *P* value of <.05 is significant.

Despite the presence of LL, both LL(+)/C and LL(+)/NC groups had deeper S3 implants than the LL(−)/NC group at both noncoronary cusp (ventricular: 19.8% ± 11.0% in LL[+]/C vs 18.5% ± 9.7% in LL[+]/NC vs 17.5% ± 12.1% in LL[−]/NC; *P* = .008) and left-coronary cusp (ventricular: 16.3% ± 11.5% in LL[+]/C vs 15.6% ± 11.1% in LL[+]/NC vs 13.9% ± 13.5% in LL[−]/NC; *P* = .020). However, there were no differences across groups in intraprocedural PVL (*P* = .65), transvalvular aortic regurgitation (*P* = .67), or mean gradient on echocardiography (*P* = .75).

### In-hospital clinical and echocardiographic outcomes

Table 4 lists the in-hospital outcomes. The overall in-hospital mortality and stroke rates were 0.9% and 1.4%, respectively, with no differences across the 4 groups. There were no differences in major vascular complications (*P* = .65) or permanent pacemaker implantation (*P* = .37) across the 3 main groups. There were no differences in the median length of stay, and 92.0% of patients were discharged home, with no difference across groups.

**Table 4:** In-hospital outcomes among LL groups in SAPIEN 3 transcatheter aortic valve replacement.

Outcome	Total (N = 1130)	LL(+)/C (n = 503)	LL(+)/NC (n = 229)	LL(−)C/NC (n = 386)	*P* (3 groups)^b^	LL(−)/C (n = 12)	*P* (4 groups)^b^
In-hospital							
Mortality	10 (0.9)	3 (0.6)	2 (0.9)	5 (1.3)	0.55	0 (0.0)	0.72
Stroke	16 (1.4)	9 (1.8)	3 (1.3)	4 (1.0)	0.64	0 (0.0)	0.78
Major vascular complication	14 (1.2)	8 (1.6)	2 (0.9)	4 (1.0)	0.65	0 (0.0)	0.79
New LBBB^a^	100 (9.8)	48 (10.5)	22 (10.8)	30 (8.6)	0.59	0 (0.0)	0.54
Permanent pacemaker^a^	75 (7.4)	28 (6.1)	19 (9.3)	24 (6.9)	0.37	4 (40.4)	0.001^b^
Median LOS, d	1	1	1	1		1	
Home discharge	1040 (92.0)	463 (92.0)	208 (90.8)	359 (93.0)	0.62	10 (83.3)	0.53
Echocardiogram at discharge							
Paravalvular leak, %					0.30		0.046^b^
None/trace	91.8	91.8	93.0	91.2		83.3	
Mild	8.1	8.2	6.6	8.8		16.7	
≥Moderate	0.1	0.0	0.4	0.0		0.0	
Transvalvular AR, %					0.79		0.93
None/trace	99.7	99.8	99.5	99.7		100.0	
Mild	0.3	0.2	0.5	0.3		0.0	
≥Moderate	0.0	0.0	0.0	0.0		0.0	
Mean gradient, mm Hg	12.5 ± 4.8	12.2 ± 4.6	12.4 ± 4.6	12.9 ± 5.1	0.11	14.5 ± 4.6	0.089
AVA, cm^2^	1.74 ± 0.47	1.76 ± 0.46	1.78 ± 0.48	1.69 ± 0.46	0.12	1.52 ± 0.57	0.11

Values are expressed as n (%), or mean ± SD (range), or median ± IQR.

AR, aortic regurgitation; AVA, aortic valve area; LBBB, left bundle branch block; LL, lucent line; LOS, length of stay; NC, noncoaxial.

a Without previous pacemaker.

b A *P* value of <.05 is significant.

**Figure 3:**
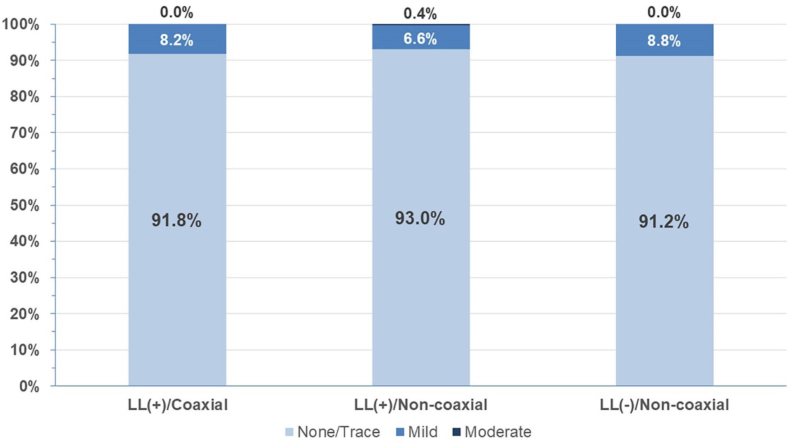
**Incidence of paravalvular leak among the 3 scenarios in SAPIEN 3/Ultra valve implantation.** There were no statistical difference (*P* = .30) in the severity of paravalvular leak on the predischarge transthoracic echocardiogram among the 3 scenarios in SAPIEN 3/Ultra implantation.

There was only 1 moderate PVL in our series at discharge, in the LL(+)/NC group, in the same patient who had two 23.0-mm S3 implanted during the same procedure. Overall incidence of mild PVL was 8.1%, with no differences across the 3 main groups (Figure 3). Mean gradient and mean aortic valve area were similar among the 3 main groups (*P* = .11 and *P* = .12, respectively). Additional analysis performed comparing deployment with dedicated LL visualization vs traditional view deployment did not show any significant difference in implant depth, coaxiality of the THV, or need for permanent pacemaker.

## Discussion

In this large-scale, single-center, retrospective study evaluating the fluoroscopic visualization of LL during S3 positioning and coaxiality of S3 valve implantation, we observe the following key findings: (1) on the 3-cusp coplanar view, LL was observed in only 64.8% of cases; (2) despite LL being present, the S3 implantation was NC in 31% of cases; (3) despite LL being present, the S3 implantation was deeper than the LL(−)/NC group; and (4) there was no impact of LL and coaxiality of S3 implantation on in-hospital clinical and echocardiographic outcomes, including pacemaker implantation, PVL, or hemodynamic performance. Our findings therefore suggest that LL visualization is not a requirement for optimal S3 implantation and has no impact on coaxiality of S3 implantation and associated outcomes (Central Illustration).

**Central Illustration:**
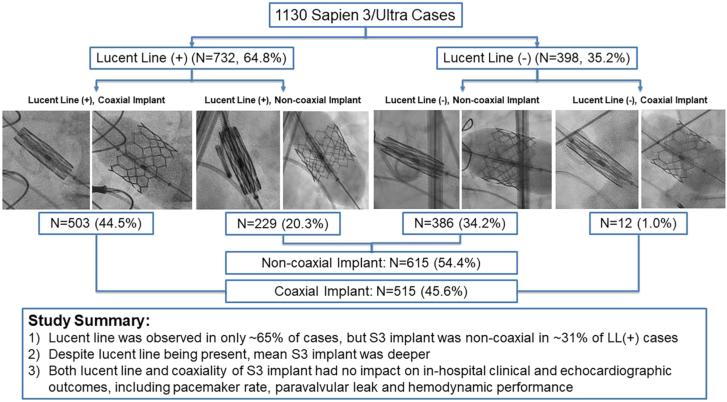
**Summary of our lucent line (LL) SAPIEN 3/Ultra TAVR study.** TAVR, transcatheter aortic valve replacement.

**Supplementary Figure 1:**
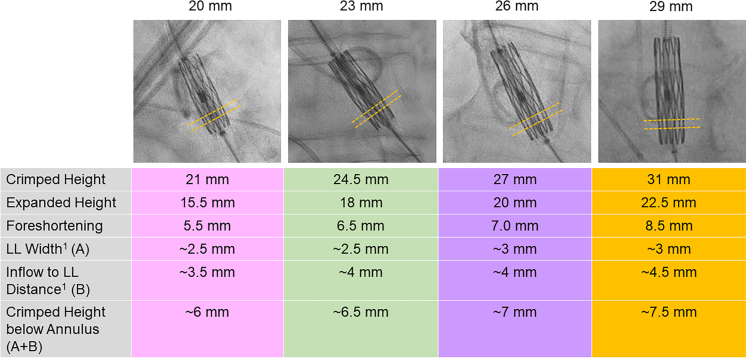


Contrary to the previous 2 studies, we for the first time identified several key observations on LL visualization during S3 implantation. First, we found that LL was present only in less than two-thirds of over 1000 S3 TAVR procedures that we have performed at the conventional deployment view. In the remaining patients, there was clearly parallax seen on the S3 valve during positioning at deployment, making LL visualization difficult if not impossible (Figure 1). Second, LL was more difficult to visualize with smaller S3 (eg, 20.0 and 23.0 mm vs 26.0 and 29.0 mm), given smaller valves have fewer number of stent frame cells crimped to form the LL. A slight parallax on a smaller S3 would make LL visualization significantly more difficult. Third, not only the crimped heights vary across S3 sizes, LL width and the S3 frame inflow-to-LL distance also appear to vary across sizes (Supplemental Figure S1). With variable foreshortening depending on the extent of S3 expansion during deployment, the final implant depth, even with LL visualization to aid positioning, may not be achieved consistently. Finally, we observed that nearly one-third of cases with LL visualized at S3 positioning ended up with a noncoaxial deployment, and these patients almost always ended up with a higher implant when the parallax of the S3 was removed during aortogram evaluation. Our study actually showed that even with LL guided deployment, those that ended up with coaxial S3 implants actually had deeper implants than those without LL during positioning and with NC implants. Therefore, our study suggests that LL visualization during S3 deployment at the conventional 3-cusp view is not always reproducible and may not be necessary to achieve a higher S3 implantation.

One potential explanation for the observed heterogeneity in our retrospective analysis of LL visualization and coaxiality of S3 implantation, is the different fluoroscopic S-curves representing the aortic annulus and the S3 THV, similar to that observed with self-expanding THVs described by Zgheib et al^8^ With S3 having a shorter frame and mounted on a flexible delivery system, the intersection of the double S-curve may be more variable to identify the optimal fluoroscopic projection, whereby both the S3 valve and annular plane would have minimal parallax to each other. The Cleveland Clinic recently reported using an RAO caudal projection to remove the parallax on the S3 valve to visualize the LL, in order to optimize valve deployment. However, this fluoroscopic projection was not achieved in the cusp-overlap view and therefore the S3 deployment likely occurred not on the annulus S-curve, leading to noncoaxial deployment relative to the annular plane.^2^ Akodad et al^4^ from Vancouver recently reported higher S3 implantation when deploying using the cusp-overlap view rather than the conventional 3-cusp view, but the authors did not report the rate of LL visualization during the cusp-overlap deployment.^4^ In fact, as a proof of concept, we rotated the C-arm from 3-cusp to cusp-overlap view during initial S3 positioning across the annulus before deployment in over 10 patients and found that LL could not be consistently visualized on the cusp-overlap view, and the final implant might not appear coaxial on cusp-overlap view (Supplemental Figure S2). We therefore abandoned attempting the cusp-overlap view to perform S3 implantation. Identifying the intersection of the double S-curve in S3 implantation for each patient may offer a reproducible approach to visualize the LL to achieve a more coaxial S3 deployment relative to the annular plane.

### Factors associated with S3 deployment on final implant depth and coaxialty

We believe that multiple factors may account for S3 implant depth and coaxiality, in addition to LL visualization. First, depending on the S3 size, the LL is located in different lengths from the S3 inflow. When positioning the LL at the annular level across all S3 sizes, one would expect a deeper implant with a 29.0-mm than 26.0- or 23.0-mm S3, given there is more stent frame length for the 29.0-mm S3 to foreshorten from the ventricular side during expansion to achieve its final implant length (Supplemental Figure S1). Therefore, one may need to position a 29.0-mm S3 more aortic than a 26.0- or 23.0-mm S3 during deployment to achieve the same implant depth.

Second, previous studies have shown the stent frame expansion of an implanted S3 depends on the degree of annular oversizing/undersizing and severity of aortic valve calcification, and frame recoil needing postdilation to better expand the S3 had been reported.^9^^,^^10^ Given S3 expands by ventricular foreshortening, an underexpanded S3 would likely have a taller frame height than a more fully expanded one. We previously published that overfilling the S3 balloon to more optimally expand and implant S3 in extremely undersized annuli did not result in excessively aortic implants but did have shallower implant depths than nominally sized annuli.^11^^,^^12^ Therefore, in addition to initial S3 positioning, the extent of annular sizing and S3 expansion during deployment would also like have an impact on the final S3 frame height and implant depth.

Third, because S3 having a shorter stent frame and a flexible delivery catheter, crossing the aortic annulus may not result in the S3 valve being coaxial to the annular plane on the 3-cusp coplanar deployment view. In fact, coaxial positioning of the S3 across the annular plane (ie, LL present) occurred in only ∼65% of cases in our study. Over 35% of cases had parallax on the S3 valve across the coplanar view, resulting in noncoaxial implant in ∼97% of the time. In addition, >30% of cases with coaxial positioning at the annulus (LL present) resulted in noncoaxial implant. These findings suggest that coaxial S3 implantation cannot be achieved consistently, despite being the S3 being coaxial at annular positioning. Further investigations will be necessary to refine delivery system design and procedural technique to aim for a more reproducible coaxial S3 positioning and implantation.

### No association between coaxiality of S3 implantation and outcomes

To the best of our knowledge, there have been no previous studies that evaluated the coaxialty of THV implantation and outcomes. Our study showed that despite S3 implantation being NC >50% of the time, there was no impact on acute procedural and in-hospital outcomes. Specifically, there were no differences in new left bundle branch block, permanent pacemaker implantation, PVL, and transvalvular gradients across the 4 comparison groups. It is therefore reassuring to see that despite achieving a NC implant in S3 TAVR, there appeared to be no short-term adverse effect on electrophysiological and hemodynamic outcomes.

### Study limitations

Our study has several limitations inherent to its retrospective design. We did not use LL to guide our S3 deployment as this was a retrospective analysis to determine the incidence of LL seen on the conventional 3-cusp deployment view. Echocardiographic evaluation was not core laboratory adjudicated, and the imager was not blinded to the intervention. Fluoroscopic evaluation of implant depth was determined by a single implanter who was also the imager (G.H.L.T.), and true implant depth would be subjected to inherent limitations of angiographic evaluation.^13^ The impact of coaxiality of S3 implantation on long-term hemodynamic performance was not determined and not a key objective of this study.

## Conclusions

LL visualization in conventional S3 TAVR could not be obtained in a significant portion of cases, did not result in a coaxial valve deployment in a majority of cases, and did not achieve a higher valve implantation. However, no short-term clinical and echocardiographic impact was observed.

## Figures and Tables

**Supplementary Figure 2:**