# Supplementary material for: Fluoroscopic “Lucent Line” Visualization in SAPIEN 3 TAVR Deployment: Reproducibility and Impacts on Outcomes
Source: J Soc Cardiovasc Angiogr Interv. 2025 Aug 19;4(9):103856. doi: 10.1016/j.jscai.2025.103856 (PMC12485540; doi:10.1016/j.jscai.2025.103856)
Supplement: Supplementary Material [file mmc1.docx]

**Supplement Data:**

There were 9 primary and 2 secondary interventional operators involved during the study period. Table below has the total number of procedures performed by each primary interventional operator during the study period.

| Primary Operator number | Number of procedures |
| --- | --- |
| Operator#1 | 442 |
| Operator#2 | 348 |
| Operator#3 | 157 |
| Operator#4 | 51 |
| Operator#5 | 50 |
| Operator#6 | 38 |
| Operator#7 | 23 |
| Operator#8 | 11 |
| Operator#9 | 10 |
